# Effectiveness of Mobile-Based Progressive and Fixed Physical Activity on Depression, Stress, Anxiety, and Quality of Life Outcomes Among Adults in South Korea: Randomized Controlled Trial

**DOI:** 10.2196/55578

**Published:** 2024-06-12

**Authors:** Ye Hoon Lee, Hyungsook Kim, Juhee Hwang, Sihyeon Noh

**Affiliations:** 1 Division of Global Sport Industry Hankuk University of Foreign Studies Gyeonggi-do Republic of Korea; 2 Department of Data Science Hanyang University Seoul Republic of Korea; 3 Hanyang Digital Healthcare Center Hanyang University Seoul Republic of Korea

**Keywords:** depressive symptoms, mental health, mobile-based exercise, non–face-to-face physical activity, progressive exercise, mobile phone

## Abstract

**Background:**

Depression acts as a significant obstacle to the overall well-being of individuals. Given the significant consequences, timely recognition and proactive steps to manage symptoms of depression become essential. Such actions not only reduce personal distress but also play a crucial role in reducing its far-reaching impact on society as a whole.

**Objective:**

In response to this concern, the objective of this study was to explore the use of mobile-based interventions as a possible remedy. More specifically, this study aimed to investigate the effectiveness of 2 types of physical activity (PA), progressive and fixed, within a mobile-based app on depression, perceived stress, anxiety, physical health, and psychological health, aiming to contribute to the optimization of mental health benefits.

**Methods:**

Participants (N=60; mean age 25.29, SD 6.10 years) were recruited using a combination of web-based and offline methods, and the study lasted for 8 weeks. The baseline and posttest questionnaires were administered to all participants. The participants were randomly assigned to 1 of the 3 groups: progressive group (n=20; performing mobile-based progressive PA), fixed group (n=20; performing mobile-based fixed intensity PA), and control group C (n=20). Data analysis involved comparing scores between the experimental and control groups using a one-way ANOVA, paired sample *t* tests (2-tailed), and repeated measures ANOVA with a 3 (group)×2 (time) design.

**Results:**

The findings revealed significant improvements in mental health indicators among participants engaged in both fixed and progressive PA groups compared with the control group. However, the fixed PA group demonstrated more significant reductions in symptoms. Specifically, the progressive PA group showed significant reductions in depression (*F*_1,36_=6.941; *P*=.01; η_p_^2^=0.16) and perceived stress (*F*_1,36_=5.47; *P*=.03; η_p_^2^=0.13), while the fixed PA group exhibited significant reductions in depression (*F*_1,37_=5.36; *P*=.03; η_p_^2^=0.12), perceived stress (*F*_1,37_=7.81; *P*=.008; η_p_^2^=0.17), and general anxiety disorder (*F*_1,37_=5.45; *P*=.03; η_p_^2^=0.13) compared with the control group.

**Conclusions:**

This study underscores the potential of mobile-based PA in improving mental health outcomes. The findings offer significant insights for mental health professionals and researchers aiming to optimize mental well-being through innovative mobile therapies.

**Trial Registration:**

Clinical Research Information Service KCT0009100; https://tinyurl.com/mr33fmur

## Introduction

### Background

Depressive disorder, commonly known as depression, is a widespread mental condition characterized by persistent feelings of sadness, loss of interest or pleasure in activities, and prolonged symptom duration [1]. Unlike normal mood fluctuations, depression significantly impairs an individual’s ability to function effectively in daily life, affecting relationships, work, and engagement in once-enjoyed activities. It can also affect concentration, memory, decision-making, and motivation, making simple tasks challenging [2]. Notably, approximately 280 million people worldwide experience depression, with a prevalence of 3.8% in the population, including 5% of adults (4% in men and 6% in women) and 5.7% among adults aged ≥60 years [3,4]. Consequently, this creates an economic burden, resulting in annual losses of approximately US $36.6 billion [5]. South Korea, which has the greatest suicide rate among Organisation for Economic Co-operation and Development countries, is a nation that encounters unique challenges in this regard. Depression rates in Korea are increasing annually, and the annual social and economic costs associated with suicide, which include the potential reduction in income, amount to approximately ₩ 4.83 trillion or KRW 4.83 trillion (equivalent to US $3.67 billion) [6]. These figures emphasize the critical nature of directing national focus toward the assessment and treatment of depression. Despite the availability of effective treatments for mental disorders, inadequate investment in mental health care, limited availability of trained health care professionals, and social stigma surrounding mental disorders contribute to barriers that impede access to effective care [7,8].

Physical activity (PA) is widely recognized as a significant contributor to mental well-being and a protective factor against depressive symptoms [9-15]. Regular participation in PA not only decreases the frequency and intensity of health problems and chronic illnesses but also improves the general standard of living, playing a vital role in maintaining good health [16]. The underlying mechanisms of the beneficial impacts of PA on depression can be outlined as follows: first, PA stimulates the secretion of endorphins, which are natural substances that elevate mood, relieve pain and tension, and induce a state of relaxation and overall contentment, and it also plays a vital function in the regulation of neurotransmitters, including serotonin, dopamine, and norepinephrine. These neurotransmitters are essential for regulating mood and emotions, ultimately contributing to enhanced mental well-being. Second, PA also serves as a means of alleviating stress, leading to a decrease in overall stress levels and enhancing persons’ ability to effectively manage daily obstacles. Third, regular PA not only improves physical fitness and body image but also boosts self-esteem and self-confidence, which in turn has the potential to decrease depressive symptoms. Fourth, PA has been linked to better sleep quality and can help regulate sleep patterns, addressing one of the common issues faced by those with depression [17-24]. Thus, regular PA can alleviate the symptoms of depression and improve overall quality of life by addressing various aspects of human functioning. Indeed, the body of scientific research supporting the benefits of PA continues to expand, highlighting its positive effects on mental health recovery and enhancement [9-16]. For instance, Marques et al [9] conducted a 4-year follow-up analysis on 32,392 European adults from 14 countries and revealed that participating in moderate or vigorous PA was linked to reduced depression scores in both genders. This association remains significant even when accounting for self-rated health, sociodemographic factors, and chronic diseases, indicating a negative correlation between PA and depression symptoms. Even a modest frequency of PA, such as twice a week, was found to lead to cumulative benefits [25].

Despite the recognized importance of PA in mental health recovery, significant limitations to participating in PA exist in traditional face-to-face PA. These limitations, which revolve around accessibility issues, create barriers that hinder people’s engagement in PA [26-28]. One aspect of accessibility is psychological distance, which encompasses factors such as a lack of interest, lack of perseverance, and limited PA knowledge. These psychological barriers contribute to individuals feeling distant from engaging in PA, reducing their motivation to participate [28-30]. Aside from psychological barriers, geographical distance poses a hindrance to involvement. Proximity to PA facilities and the timing of PA programs can significantly impact convenience for participants, adding to the psychological distance they experience [27]. Face-to-face programs typically operate on fixed schedules, which may not align with everyone’s availability. This lack of flexibility also poses challenges for individuals with busy lifestyles or conflicting commitments, making it difficult for them to commit to specific classes or training sessions. The financial aspects further emphasize the constraints of face-to-face PA programs. Face-to-face PA programs typically involve additional expenses, such as fees for gym memberships, classes, or personal training. These financial responsibilities can serve as obstacles, especially for individuals with constrained budgets or restricted financial means [31]. Collectively, these factors serve as obstacles to face-to-face PA engagement, hindering persons from experiencing the advantages of PA for mental health improvement and enhancement. Considering these limitations, it is crucial to identify alternative choices that provide enhanced accessibility, flexibility, and affordability to ensure that individuals participate in PA and benefit from its favorable effects on their mental well-being.

Mobile-based PA programs are structured interventions that use mobile technology, such as smartphones, tablets, wearable activity trackers, and PDAs. These programs deliver personalized plans for PA, including aerobic exercises, strength training, and flexibility routines. They also incorporate real-time monitoring and tracking features to assess participants’ progress [32]. Compared with traditional face-to-face PA approaches, mobile-based PA programs offer several advantages. First, it tackles the accessibility concerns by enabling individuals to participate in PA at any time and in any location, thus eliminating the limitations of time and place that are associated with face-to-face PA. Studies indicate that mobile-based programs can successfully encourage participation in PA without the requirement of physical gym facilities [33]. In addition, the use of mobile technology allows mobile-based PA programs to provide systematic supervision through customized workout plans and tailored PA activities that cater to the specific demands and fitness levels of individuals [34]. This process offers them prompt feedback and encouragement to adhere to their fitness objectives. Finally, the use of mobile PA apps provides a cost-efficient method for reducing expenses connected to facility use and health concerns [35] while it significantly improves physical health variables, such as managing weight; self-confidence; and mental health aspects, such as decreasing depression, stress, and increasing enjoyment of life [36-41]. For example, Murray et al [39] found that college students who engaged in approximately 150 minutes of moderate to vigorous PA per week over an 8-week period showed significant decreases in depression and anxiety. In general, the mobile-based PA program offers the ability to overcome the restrictions of accessibility, cost, and interest that are commonly associated with in-person PA.

Recognizing the beneficial effects of mobile-based PA programs on mental well-being, it is imperative to further investigate and analyze the impact of different types of PA on mental health. One of the fundamental principles of fitness training is progressive exercise, which involves a gradual increase in the intensity, duration, or complexity of the exercise program over time. The objective of progressive exercises is to continually challenge the body and induce adaptation, leading to improvements in strength, endurance, flexibility, and other desired fitness outcomes [16]. While this type of PA has proven to effectively enhance physical health among individuals, research has also demonstrated the effectiveness of progressive PA in affecting various indicators of mental health [42,43]. For example, Singh et al [42] conducted a 10-week randomized controlled trial involving older adults aged ≥60 years and found that progressive resistance training significantly reduced depression measures. In addition to its impact on the symptoms of depression, previous literature has revealed that individuals engaging in regular PA, including progressive PA, experienced improved mood, well-being, and quality of life compared with those with sedentary lifestyles [43]. These studies offer compelling evidence supporting the positive effects of progressive PA on mental well-being, encompassing reductions in depressive and anxiety symptoms, improvements in mood, and an overall enhancement of quality of life. While research has already demonstrated the benefits of progressive PA in traditional settings, there remains a need for further investigation to explore its effectiveness and implementation in the context of mobile-based PA. Remarkably, no studies have specifically examined the application of progressive exercise principles within a mobile-based PA framework. Understanding the potential impact of progressive PA within mobile-based interventions could have substantial implications for promoting mental health and well-being in an increasingly digitally connected world.

### Objective

In summary, previous studies have successfully demonstrated that regular PA has a positive impact on alleviating mental issues such as depression, anxiety, and stress and improving the quality of life. However, it is important to note that most previous research has predominantly focused on face-to-face PA interventions or mobile-based PA characterized by a fixed intensity. Unfortunately, limited studies have ventured into exploring the potential benefits of mobile-based PA through apps or the implementations of progressive PA approaches. To address this research gap, this study aimed to investigate the effects of both progressive and fixed PA interventions, using a mobile app, on depression, stress, anxiety, and quality of life of adults in South Korea. We hypothesized that both progressive and fixed PA interventions could lead to improvements in mental health indicators such as depression, perceived stress, and anxiety, as well as enhance quality of life indicators related to physical and psychological health. Through this exploration of the benefits of mobile-based PA programs and their potential to positively influence mental health outcomes, we aimed to have a deeper understanding of how technology-enabled interventions can play a significant role in promoting well-being in modern society.

## Methods

### Participants

The recruitment of participants was meticulously designed to ensure a representative and diverse sample. In order to do this, a blend of web-based and offline recruitment techniques was used, with each method playing a role in the random sampling procedure. Web-based recruitment efforts targeted a diverse pool of potential participants by leveraging various digital platforms such as social media groups, forums, and university websites. Our objective was to attract individuals with diverse backgrounds who were interested in contributing to mental health research. Offline recruitment strategies complemented the web-based efforts by reaching individuals who may not have regular access to internet resources. Promotional materials were disseminated in public venues such as university counseling centers, local community centers, and coffee shops. These venues were selected to ensure visibility and accessibility to individuals lacking access to internet platforms. By using a combination of web-based and offline recruitment techniques, we aimed to create a sample that reflects the diversity of the target population. The recruitment period lasted from the first to the fourth week of March 2023, and both web-based and offline advertisements were used. The participants completed the baseline and posttest questionnaires in the first weeks of April and June 2023, respectively.

Eligibility criteria were predetermined; participants should be (1) healthy adults, aged between 18 and 65 years to ensure a sample representative of the adult population; (2) fluent in reading, writing, and conversing in Korean to ensure participants could fully understand and engage with study materials and instructions; (3) having no contraindications for PA as assessed by the Physical Activity Readiness Questionnaire (PAR-Q) [44] to ensure participant safety during the intervention period; (4) willing to be randomized into an intervention that may require up to 2 hours per week of their time to ensure participant commitment and adherence to the study protocol; (5) familiar with using a smart mobile phone to ensure participants could adequately perceive and respond to visual and auditory stimuli presented during the study; (6) having normal (or corrected-to-normal) vision and hearing; and (7) having no history of psychosis to minimize potential risks associated with mental health conditions during the intervention period.

### Sample Size Calculation

In this study, the appropriate sample size was determined using the G* power calculator (version 3.1.9.4; Heinrich-Heine-Universität) [45]. The aim was to achieve a significance level of .05 and a statistical power of 95%, with an effect size of 0.36, based on the data from a prior study that investigated the impact of mobile-based PA interventions on depression [41]. The calculations indicated that 30 participants were required to reach the desired power of 0.95. Accounting for a 57% dropout rate observed in computer-based psychological treatment [46], the total sample size needed was calculated to be 47 participants to maintain a power of 0.95. Therefore, including 60 participants was anticipated to provide sufficient statistical power for the study.

### Procedure

Initially, a total of 60 participants were recruited. Following the fulfillment of inclusion criteria, the process of participant allocation used a simple block randomization methodology. This approach involved using the resources available on the open-source website to assign a total of 60 participants into 3 distinct groups: the progressive group, the fixed group, and the control group. Each group comprised 20 participants. The procedure involved the creation of 10 blocks, each containing 6 participants, as the designated units for randomization. Participants were allocated to the 3 groups within each block using randomization, guaranteeing an equal distribution of participants among the groups throughout each block. At first, the groups had the following gender distribution: the progressive group consisted of 10 men and 10 women, the fixed group consisted of 14 men and 6 women, and the control group consisted of 12 men and 8 women. After the random selection, 2 women from the fixed group expressed a preference for the control group due to their disinclination for PA, while 2 men from control group wished to switch to the fixed group in order to participate in PA. Consequently, the research protocol allowed these participants to transfer between groups, leading to the following revised composition: the progressive group consisted of 10 men and 10 women, the fixed group consisted of 16 men and 4 women, and the control group comprised 10 men and 10 women.

After the randomization, the researchers contacted the participants via the Webex platform (Cisco) for both baseline and posttest evaluations at baseline and 8 weeks. Initially, the participants were instructed to fill out the web-based questionnaire, which encompassed an explanation of the study’s objectives, privacy safeguards, and procedures to ensure confidentiality during the baseline evaluation, informed consent, and Physical Activity Readiness Questionnaire (PAR-Q) [44]. During the intervention phase, the researchers consistently spoke with the participants on a weekly basis to ensure that they followed the instructions and to track their advancement. They supervised the completion of the assigned PA and provided assistance for any personal queries. To validate the participants’ activities, the researchers received verification photos via messenger, showing the number, duration, and type of PA sessions for each week. To uphold consistency and anonymity, all surveys were collected without personal identifiers, and each participant used a unique ID for completing the surveys during both the baseline and postevaluation phases.

### Ethical Considerations

This study received ethical approval from the institutional review board of Hankuk University of Foreign Studies (approval number HIRB-202306-HR-001). Before commencing the study, all necessary approvals were obtained to ensure compliance with ethical standards and the protection of participants’ rights. The objectives and methods of the study were clearly explained to potential participants, and only those who provided written informed consent and willingly chose to participate were included.

### Instrument

#### Depression

Depression was measured using the Korean version of the Patient Health Questionnaire-9 (PHQ-9). The PHQ-9 has been reported to be a reliable and valid tool for screening and assessing depressive symptoms [47]. The inventory has 9 items pertaining to symptoms of depression, including diminished interest or pleasure, mood fluctuations, disruptions in sleep patterns, exhaustion, and alterations in food behavior. The scoring system ranges from 0 (not at all) to 3 (nearly every day) for each item. A greater cumulative score on the PHQ-9 signifies a higher level of depression symptoms, whereas a lower score signifies a lower level of symptoms. The Cronbach α coefficients were 0.86 for the study sample and 0.81 in the initial validation [47].

#### Perceived Stress

The assessment of perceived stress was conducted by using the Brief Encounter Psychosocial Instrument-Korean version (BEPSI-K). BEPSI-K is a tool that has been created using the dynamic interaction model of stress and its detrimental effects on health [48]. This is a Korean version of the original BEPSI that has been tested and confirmed to be reliable and legitimate [49]. The scoring system for each item is based on a scale that spans from 1 (not at all) to 5 (always). Consequently, elevated scores on the BEPSI-K signify an increased perception of stress, whereas lower scores indicate a decreased experience of stress. In our investigation, the Cronbach α coefficient obtained from our sample was 0.81, which is somewhat higher than the value of 0.80 reported in the original validation [48].

#### Anxiety

We used the Generalized Anxiety Disorder-7 (GAD-7) to evaluate the symptoms associated with generalized anxiety disorder. The GAD-7 is beneficial because of its simplicity and limited number of components, which allows for easy access [50]. The scale has 7 items that evaluate anxiety symptoms. Each item is rated on a scale ranging from 0 (not at all) to 3 (nearly every day). The GAD-7 scores are directly proportional to the frequency and severity of symptoms associated with generalized anxiety disorder. Higher scores indicate a greater number and intensity of symptoms, whereas lower scores indicate a lesser number of symptoms. The Cronbach α coefficient in our study sample was 0.90, although the original validation study reported a value of 0.92 [50].

#### Quality of Life

The study used the Korean version of the World Health Organization Quality of Life-Brief assessment questionnaire to measure the quality of life. The World Health Organization Quality of Life-Brief is a reliable and valid tool developed by the WHO in 1996 as a shortened version of the WHOQOL-100, which scientifically measures and evaluates the quality of life [51]. It comprises 5 domains: physical health, psychological health, social relationships, environmental factors, and overall quality of life. This study used 13 items, including 7 items related to physical health and 6 items related to psychological health. These items assess various aspects, such as physical satisfaction, need for treatment, sleep quality, and negative emotions. Scores range from 1 (not at all) to 5 (very much)***.*** The Cronbach α values were 0.79 (physical health) and 0.81 (psychological health) for the study sample, and 0.71 (physical health) and 0.83 (psychological health) in the original validation [51]. In our study sample, the Cronbach α values were 0.79 for physical health and 0.81 for psychological health, while the original validation reported values of 0.71 for physical health and 0.83 for psychological health.

### Mobile-Based PA Program

The program used in this study is a personalized health care app that provides artificial intelligence–based wellness services in the areas of PA, diet, nutrient, and mental health. The app offers various PA videos tailored to different goals including 32 stretching movements, 44 full-body aerobic PA, 20 upper-body strengthening PA, and 18 lower-body strengthening PA, categorized according to difficulty (easy, moderate, and difficult). Thus, participants could select desired movements and adjust the number of sets to create personalized PA lists. All participants, irrespective of their group assignment, were granted equal access to the complete app. They were provided the freedom to choose and combine PA according to their individual preferences and needs and had the flexibility to adjust the number of sets as desired.

In the context of this app-driven intervention, participants in both the progressive and fixed groups were instructed to engage in PA for 8 weeks, with a minimum of 3 sessions per week and a minimum PA duration of 15 minutes per day. For the progressive group, participants were guided to participate in progressive PA, which involved a daily and weekly gradual increase in difficulty level, duration, and intensity. For example, the daily program of the progressive group mostly involved performing 2 to 3 movements at each difficulty level, with the number of repetitions increasing as the difficulty level increased. That is, the routine started with easier movements and became more difficult over time, with more repeats and higher levels of difficulty during each session. Furthermore, the progressive group performed 12 sets in weeks 1 to 2, a total of 18 sets in weeks 3 to 4, a total of 27 sets in weeks 5 to 6, and 36 sets in weeks 7 to 8. Finally, the progressive group was encouraged to gradually increase the duration of PA, starting with a minimum of 15 minutes in the first week and progressing up to approximately 30 minutes by the eighth week. This methodology facilitated a seamless transition toward progressive PA engagement.

The fixed group in our study followed a different approach compared with the progressive group. Instead of experiencing a gradual increase in intensity, frequency, duration, and difficulty levels, participants in the fixed group engaged in a consistent and predetermined intensity level and set of PA throughout the 8-week period. As for the intensity levels, they were guided to choose and proceed with a moderate intensity PA in the app. The participants freely chose and performed 6 types of PAs that they desired at the specified intensity level. They also performed the PA with a fixed number of sets and repetitions for each session, maintaining a PA frequency of approximately 16 to 17 minutes per session, 3 times a week, from the first to the eighth week. This steady and consistent PA routine was maintained without the progressive changes in difficulty levels and repetition experienced by the progressive group. In contrast, the control group did not participate in any structured PA during the study period.

### Covariate

In this study, gender was incorporated as a covariate variable in the statistical analysis to consider its potential impact on mental health outcomes, such as depression, anxiety, and perceived stress. Research has consistently demonstrated gender differences in the occurrence, presentation, and management of mental health disorders [52,53]. By controlling for gender, we aimed to mitigate the potential confounding effects of gender-related factors on the study outcomes, thereby enhancing the internal validity of the expected findings. Furthermore, by implementing gender control, we aimed to distinguish the specific impacts of the independent variable, specifically, the PA intervention type, on the dependent variables, facilitating more accurate conclusions from the findings.

### Data Analysis

Initially, we conducted descriptive analysis to obtain frequencies and percentages for categorical variables and means and SDs for continuous variables. Subsequently, we assessed differences between groups at baseline and posttest evaluations by using a one-way ANOVA for continuous variables and a chi-square test for categorical variables. When we found statistically significant differences between the groups through the initial one-way ANOVA, we proceeded with post hoc tests for further analysis. We used the appropriate Tukey honestly significant difference test. By performing post hoc tests, we aimed to explore and interpret the significant differences observed during the initial analysis, contributing to a more nuanced interpretation of the study results. To evaluate the effects of the mobile-based PA, we compared and analyzed self-report questionnaire scores between the experimental and control groups using SPSS Statistics for Windows (version 22.0; IBM Corp). Initially, we conducted paired sample *t* tests within each group to compare the baseline and postevaluation measurements separately for both groups. Subsequently, we performed a 3 (group)×2 (time) repeated measures ANOVA (RMANOVA) using baseline and postevaluation measurements, with the group as the independent variable. To control for their influence on the study outcomes [52], we included gender as a covariate in our analysis. Upon finding significant differences in the 3×2 RMANOVA, we further examined the individual group comparisons using a 2 (group)×2 (time) RMANOVA. Specifically, we compared the progressive PA group with the control group, as well as the fixed PA group with the control group, to identify which specific group differences contributed to the observed effects.

## Results

### Demographic Characteristics at Baseline

**Figure 1:**
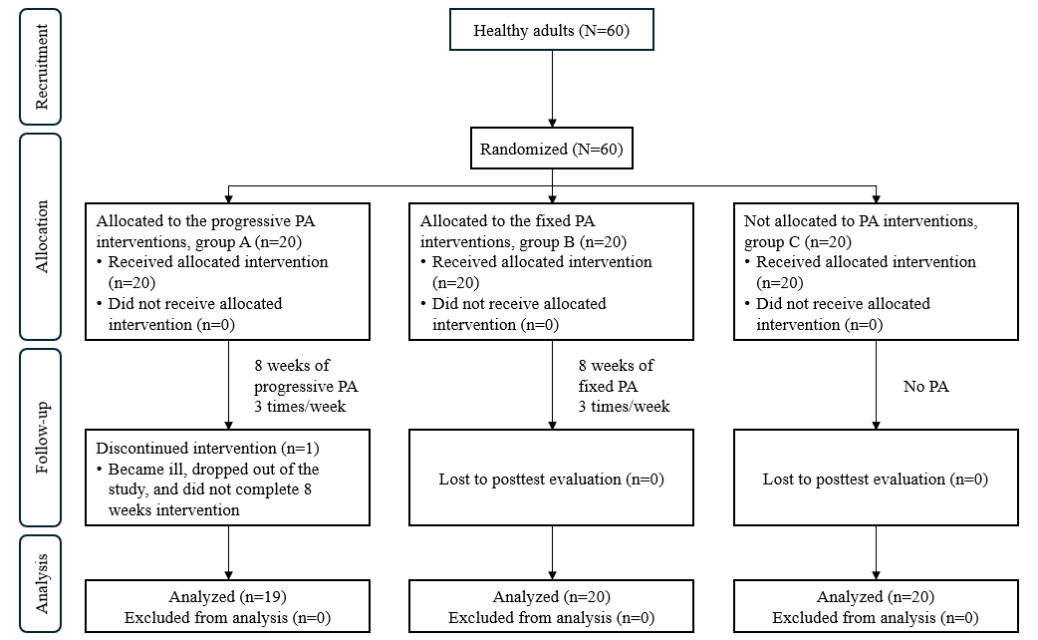
Study flow diagram. PA: physical activity.

Figure 1 illustrates the CONSORT (Consolidated Standards of Reporting Trials) diagram, presenting the participant flow throughout the study. The initial 60 participants were divided into 3 groups: progressive group, fixed group, and control group. The progressive group followed a mobile-based progressive PA program, with a daily and weekly gradual increase in the intensity and difficulty levels. More specifically, 20 (33%) participants were assigned to each of the experimental groups A and B, while 20 (33%) participants were allocated to the control group C. Notably, 98% (39/40) of the participants in progressive and fixed groups and 100% (20/20) of the participants in the control group completed the posttest assessment.

In this study, a comprehensive statistical analysis was performed to investigate potential demographic differences between the study groups. The results indicated no statistically significant variations in gender (*P*=.10), age (*P*=.78), height (*P*=.06), weight (*P*=.15), BMI (*P*=.31), and internet self-efficacy (*P*=.58) among the groups. In addition, the level of internet self-efficacy remained consistent throughout the various study groups. Table 1 displays the demographic attributes of the participants.

**Table 1:** Demographic characteristics.

Characteristics				Progressive group (n=19)		Fixed group (n=20)		Control group (n=20)		*P* value	
**Categorical variables, n (%)**											
	**Gender**										.10
		Man	10 (53)		16 (80)		10 (50)				
		Woman	9 (47)		4 (20)		10 (50)				
	**Education**										.51
		High-school graduation	1 (5)		1 (5)		4 (20)				
		College students	12 (63)		13 (65)		11 (55)				
		Bachelor’s degree	4 (21)		5 (25)		5 (25)				
		Graduate degree	2 (11)		1 (5)		0 (0)				
	**Experience in mobile-based physical activity app**										.10
		Yes	10 (53)		4 (20)		7 (35)				
		No	9 (47)		16 (80)		13 (65)				
**Continuous variables, mean (SD)**											
	Age (y)		25.11 (4.62)		26.05 (6.28)		24.7 (7.28)		.78		
	Height (cm)		170.61 (7.82)		172.52 (6.01)		167.00 (7.28)		.06		
	Weight (kg)		70.88 (13.35)		70.14 (15.85)		63.47 (14.42)		.15		
	BMI (kg/m^2^)		24.50 (3.80)		22.27 (6.58)		22.44 (3.90)		.31		
	Internet self-efficacy		4.00 (1.20)		3.65 (1.39)		4.05 (1.36)		.58		

### Differences Observed in the 3 Groups

This study used a one-way ANOVA to examine the variables of depression, stress, anxiety, and physical and psychological health among 3 unique groups at both the baseline and postevaluation stages. The Levene test was conducted to assess whether the variances were equal across the 3 groups (PHQ-9, *P*=.92; BEPSI-K, *P*=.57; GAD-7, *P*=.70; physical health, *P*=.97; and psychological health, *P*=.51). Therefore, there was no significant evidence of a difference in variances among the groups at the baseline evaluation.

The characteristics of the participants in the progressive group (n=19), fixed group (n=20), and control group C (n=20) at baseline are summarized in Table 2. The results revealed that no statistically significant differences existed between the 3 groups for any of the variables at baseline.

Table 3 provides a summary of the intergroup comparisons following the intervention. The analysis revealed statistically significant differences between groups as determined by one-way ANOVA for depression (*F*_2,56_=5.06; *P*=.01; η_p_^2^=0.15) and GAD-7 (*F*_2,56_=4.13; *P*=.02; η_p_^2^=0.12). A Tukey post hoc test revealed significant differences between the progressive group and control group for depression (mean difference −3.27; *P*=.01) and for GAD-7 (mean difference −0.33; *P*=.03). There was no statistically significant difference between the groups for perceived stress (*F*_2,56_=3.12; *P*=.05), physical health (*F*_2,56_=3.08; *P*=.05), and psychological health (*F*_2,56_=1.04; *P*=.36).

**Table 2:** Baseline evaluation.

	Progressive group (n=19), mean (SD)	Fixed group (n=20), mean (SD)	Control group (n=20), mean (SD)	*F* test (*df*)	*P* value	Cohen *d*
PHQ-9^a^	4.15 (4.21)	4.75 (4.27)	4.85 (4.62)	0.14 (57)	.87	0.004
BEPSI-K^b^	1.65 (0.66)	1.83 (0.57)	1.72 (0.53)	0.44 (57)	.64	0.015
GAD-7^c^	0.33 (0.45)	0.45 (0.52)	0.44 (0.60)	0.29 (57)	.75	0.010
WHOQOL^d^—physical health	3.11 (0.46)	2.80 (0.48)	2.77 (0.50)	2.83 (57)	.07	0.091
WHOQOL—psychological health	3.37 (0.49)	3.11 (0.62)	3.15 (0.59)	1.18 (57)	.31	0.040

^a^PHQ-9: Patient Health Questionnaire-9.

^b^BEPSI-K: Brief Encounter Psychosocial Instrument-Korean version.

^c^GAD-7: Generalized Anxiety Disorder-7.

^d^WHOQOL: World Health Organization Quality of Life.

**Table 3:** Posttest evaluation.

	Progressive group (n=19), mean (SE)	Fixed group (n=20), mean (SE)	Control group (n=20), mean (SE)	Progressive vs control group, GMD^a^ (95% CI)	Fixed vs control group, GMD (95% CI)	Progressive vs fixed group, GMD (95% CI)
PHQ-9^b^	1.52 (1.61)	2.25 (1.97)	4.80 (5.24)	−3.27 (−5.88 to −0.65)^c^	−2.55 (−5.13 to 0.03)	−0.72 (−3.33 to 1.89)
BEPSI-K^d^	1.41 (0.42)	1.39 (0.39)	1.68 (0.86)	−0.39 (−0.84 to 0.06)	−0.41 (−0.85 to 0.03)	0.02 (−0.42 to 0.47)
GAD-7^e^	0.16 (0.17)	0.19 (0.25)	0.50 (0.63)	−0.33 (−0.64 to −0.02)^c^	−0.31 (−0.61 to −0.02)	−0.03 (−0.34 to 0.28)
WHOQOL^f^—physical health	3.12 (0.53)	2.72 (0.48)	2.95 (0.54)	0.39 (−.01 to 0.71)	0.30 (−0.09 to 0.70)	0.09 (−0.31 to 0.50)
WHOQOL—psychological health	3.40 (0.52)	3.28 (0.63)	3.11 (0.69)	0.29 (−0.36 to 0.60)	0.17 (−0.30 to 0.64)	0.09 (−0.32 to 0.50)

^a^GMD: group mean difference.

^b^PHQ-9: Patient Health Questionnaire-9.

^c^*P*=.009 for Patient Health Questionnaire-9 and *P*=.02 for Generalized Anxiety Disorder-7.

^d^BEPSI-K: Brief Encounter Psychosocial Instrument-Korean version.

^e^GAD-7: Generalized Anxiety Disorder-7.

^f^WHOQOL: World Health Organization Quality of Life.

### Effectiveness of Mobile-Based PA on the Outcomes

**Figure 2:**
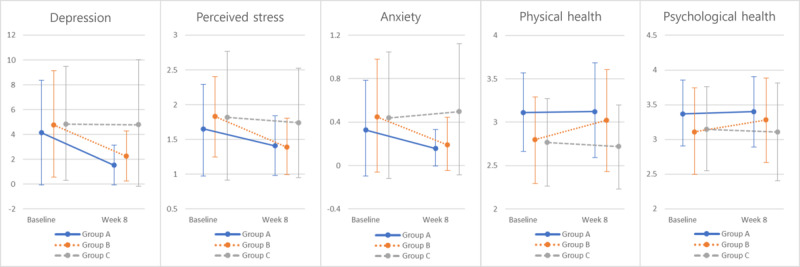
The results of the interactional analysis.

Table 4 and Figure 2 illustrate the changes observed in the experiment and control groups from baseline to program completion.

There was a significant positive interaction found between group and time in relation to depression (*F*_2,55_=3.76; *P*=.03; η_p_^2^=0.12) and perceived stress (*F*_2,55_=4.77; *P*=.01; η_p_^2^=0.15). These results suggest that depression and perceived stress levels changed over time, and the impact of the intervention was dependent on the group assignment. However, no significant interaction was observed for GAD-7 (*F*_2,55_=2.67; *P*=.08; η_p_^2^=0.09), physical health (*F*_2,55_=1.72; *P*=.19; η_p_^2^=0.06), and psychological health (*F*_2,55_=1.48; *P*=.24; η_p_^2^=0.05) between the groups. Specifically, the post hoc analysis showed a significant difference in the change of depression (*F*_1,36_=6.94; *P*=.01; η_p_^2^=0.16) and perceived stress (*F*_1,36_=5.47; *P*=.02; η_p_^2^=0.13) between progressive group and control group over time. Further, a significant difference in the change of depression (*F*_1,37_=5.36; *P*=.02; η_p_^2^=0.12), perceived stress (*F*_1,37_=7.81; *P*=.01; η_p_^2^=0.17), and GAD-7 (*F*_1,37_=5.45; *P*=.02; η_p_^2^=0.13) between the fixed group and control group was also observed across time.

**Table 4:** Differences in the outcome variables across time (n=59).

Outcome			Evaluation											
			Baseline, mean (SD)		Posttest, mean (SD)		Difference							
							Values, mean (95% CI)		*t* test (*df*)		*P* value		Cohen *d*	
**PHQ-9^a^**														
	Progressive	4.15 (4.21)		1.52 (1.61)		2.63 (0.52 to 4.73)		2.54 (56)		.02		0.82		
	Fixed	4.75 (4.27)		2.25 (1.97)		2.50 (0.36 to 4.63)		2.37 (56)		.02		0.75		
	Control	4.85 (4.62)		4.80 (5.24)		0.05 (−3.11 to 3.21)		0.03 (56)		.98		0.01		
**BEPSI-K^b^**														
	Progressive	1.65 (0.66)		1.41 (0.42)		0.24 (−0.12 to 0.60)		1.34 (56)		.18		0.43		
	Fixed	1.83 (0.57)		1.39 (0.39)		0.44 (0.12 to 0.76)		2.82 (56)		.008		0.90		
	Control	1.82 (0.91)		1.74 (0.78)		−0.08 (−0.52 to 0.36)		−0.36 (56)		.71		0.09		
**GAD-7^c^**														
	Progressive	0.33 (0.45)		0.16 (0.17)		0.17 (−0.05 to 0.39)		1.55 (56)		.13		0.50		
	Fixed	0.45 (0.51)		0.19 (0.25)		0.26 (0.00 to 0.52)		2.05 (56)		.04^b^		0.64		
	Control	0.44 (0.60)		0.50 (0.63)		−0.05 (−0.45 to 0.33)		−0.29 (56)		.77		0.09		
**WHOQOL^d^—physical health**														
	Progressive	3.11 (0.46)		3.12 (0.53)		−0.01 (−0.33 to 0.32)		.96 (56)		.96		0.02		
	Fixed	2.80 (0.48)		3.02 (0.56)		−0.22 (−0.56 to 0.10)		−1.37 (56)		.17		0.42		
	Control	2.77 (0.50)		2.72 (0.48)		0.05 (−0.26 to 0.37)		0.36 (56)		.72		0.10		
**WHOQOL**—**psychological health**														
	Progressive	3.37 (0.49)		3.40 (0.52)		−0.02 (−0.35 to 0.30)		.87 (56)		.87		0.06		
	Fixed	3.11 (0.62)		3.28 (0.63)		−0.16 (−0.56 to 0.23)		−0.83 (56)		.40		0.27		
	Control	3.15 (0.59)		3.11 (0.69)		0.03 (−0.38 to 0.44)		0.16 (56)		.87		0.06		

^a^PHQ-9: Patient Health Questionnaire-9.

^b^BEPSI-K: Brief Encounter Psychosocial Instrument-Korean version.

^c^GAD-7: Generalized Anxiety Disorder-7.

^d^WHOQOL: World Health Organization Quality of Life.

## Discussion

### Principal Findings

This study investigated the impact of progressive and fixed PA programs delivered through a mobile app on mental health indicators (depression, perceived stress, and anxiety) and quality of life (physical and psychological health) among South Korean adults. Following the intervention, the findings of this study indicated that a significant positive interaction effect existed between group and time for depression and perceived stress, implying that the effectiveness of the interventions in reducing the outcomes may have varied depending on the specific group in which they participated. Thus, we conducted additional statistical analyses to examine the impact of each PA type on the outcome variables in the experimental groups and compare the variables with the control group, leading to the following discussion.

First, we observed that participants in both the progressive and fixed PA groups showed a greater reduction in depression and perceived stress compared with those in the control group. Engaging in PA, including progressive PA, has been shown to stimulate the release of endorphins, which are neurotransmitters associated with feelings of well-being and happiness [17,54]. Furthermore, regular PA promotes neuronal growth and connectivity, particularly in brain regions that are involved in mood regulation, such as the hippocampus and prefrontal cortex [20,21,55]. These alterations in neurochemical and neurobiological processes may help reduce depression symptoms [54,55]. In addition, engaging in PA via a mobile app might function as a diversion from detrimental ideas and excessive contemplation, which are frequently linked to depression [56]. By prioritizing PA at home and attaining specific objectives and significant achievements, individuals can shift their focus away from depressed symptoms and cultivate a more positive mindset. As a result, those who participate in both the progressive and fixed PA groups may observe a more significant decrease in depression and perceived stress compared with those who do not engage in PA. Previous literature has found the significant impact of mobile-based PA programs on mental health [36-41], and this aligns with the findings of this study. Theoretical implications of these findings in academia endorse the use of both regular fixed PA and progressive PA through mobile apps as potential therapies for mitigating depression and stress. These findings contribute to a growing body of research on the effectiveness of mobile-based therapies in enhancing mental well-being.

Along with the finding of significant impact of both progressive and fixed PA on mental health indicators, a critical finding of this study is the superior improvement observed in the outcome variables within the fixed PA group compared with the progressive PA group. This finding is somewhat different from those of the previous literature exploring the impact of progressive and fixed PA on physical health outcomes [57-59]. However, this study found that fixed PA showed better results in terms of mental health indicators. One possible explanation could be related to motivation and adherence to the prescribed PA program. In this study, the fixed group encompassed a relatively higher proportion of men (16/20, 80%) compared with that in the other groups (control and progressive groups: 10/20, 50%), while 2 men from the control group switched to the fixed group, suggesting their high motivation for fixed PA engagement. Previous literature has consistently reported a positive association between motivation and mental well-being [60,61]. For example, a meta-analysis on student motivation [60] identified that amotivation (ie, lack of motivation) was positively associated with depression and anxiety, whereas regulation (ie, self-directed value-driven motivation) and intrinsic motivation were negatively associated with only anxiety. This aligns with the result of this study. Furthermore, Lauderdale et al [62] noted that male college students had more intrinsic motivations than women college students. Thus, it is possible that the fixed group includes participants with higher motivation than the progressive group, which in turn demonstrates better improvement in mental health outcomes, specifically with regard to anxiety.

Another possible explanation may be related to the psychological and physical efforts incurred during the intervention period. The progressive PA group may have faced challenges related to the increasing complexity and intensity of their PA program over time. Some participants might have been reluctant to increase the duration and intensity but felt obligations to adhere to the prescribed protocol due to their commitments to the research protocol. Furthermore, engaging in approximately 30 minutes of PA at weeks 7 and 8 could have induced more physical exertion and stress in the progressive group compared with the fixed group who engaged in approximately 17 minutes of PA. Indeed, grounded upon the reversal theory [63], Kerr and Kuk [64] found that compared with running at a low intensity, running at a high intensity resulted in significantly higher scores for physical stress and exertion among participants who ran 1.7 km. Thus, the gradual progression and high-intensity PA could have led to psychological and physical strain, potentially affecting their mental conditions and generating conflicts with the mental health enhancement. In contrast, participants in the fixed PA group may have experienced less or no psychological strain due to the consistent and predictable nature of their PA protocols. Knowing exactly what to expect in terms of PA duration, intensity, and routine may have provided a sense of structure and control, which may lead to psychological comfort during the intervention phase. Thus, in this context, although progressive PA still had significant impacts on mental health outcomes, the effect may not have been as pronounced as the effect in the fixed group, which did not incur psychological strains and emotional challenges related to engaging in PA at different and higher intensities and durations. It is important to note that this interpretation is only speculative, and further investigation into the psychological mechanisms underlying both progressive and fixed PA protocols on anxiety is warranted. In addition, although the progressive group did not demonstrate statistically significant reductions in anxiety during the 8-week intervention period, it is worth noting that the paired sample *t* test revealed a decrease in the anxiety level from 0.33 to 0.16 (*P*=.13). This suggests a potential trend toward improvement in anxiety within the progressive group, albeit not reaching statistical significance.

Although this study found that the fixed PA group showed better results in mental health outcomes than the progressive PA group, the main advantage of this study is the demonstration of the efficacy of a mobile-based progressive PA program in diminishing different mental health markers, such as depressive symptoms and perceived stress. Notably, this study is the first to apply the principle of progressive exercise in the mobile PA app context. Prior studies have primarily explored the impact of progressive PA on mental health [42,43] and overall health indicators [57-59] within traditional face-to-face PA contexts. In this study, the researchers expanded the research paradigm by introducing the principles of progressive exercise into the mobile context and specifically investigated the effects of progressive exercise principles in comparison with the control group. Thus, this study contributes significantly to our understanding of the effectiveness of different types of mobile-based PA programs in enhancing mental health. The incorporation of progressive exercise principles into mobile PA programs offers the potential and additional avenues for individuals to experience mental health benefits.

Finally, this study found that neither progressive nor fixed mobile phone–based PA programs had a significant impact on the participants’ quality of life (*P*=.19 for physical health and *P*=.24 for psychological health), as measured by physical and psychological health. While there was a slight increase in the average scores for both physical and psychological health in both progressive and fixed groups, these increases were not statistically significant. There are several possible explanations for the lack of significant results. First, the duration of the intervention might not have been sufficiently long to produce significant changes in the participants’ quality of life. Quality of life is a multifaceted construct that may require a longer period of sustained PA to observe meaningful improvements compared with the negative mental health indicators of depression, stress, and anxiety [65,66]. A meta-analysis of 16 studies examined the impact of PA on the quality of life among patients with cancer [65], and the average duration of each study was 17.18 minutes, ranging from 3 weeks to 16 months. Second, the level of adherence to the prescribed PA programs varied among the participants. Variations in adherence may have influenced the outcomes, as participants who did not consistently engage in PA may not have experienced the full potential benefits.

### Limitations and Future Research Directions

Although this study has made valuable findings, it is important to acknowledge its limitations. First, deviation from the initial randomization strategy creates intricacies that may impact the internal validity of the investigation. Although participant choice and ethical responsiveness are prioritized, it is important to acknowledge that these transfers could introduce confounding variables that may affect the results of the study. For example, a higher proportion of men in the fixed group than that in the progressive group, along with the transfer of men to the fixed group, might lead to differences in motivation levels between the groups. This could possibly result in bias in favor of the fixed group over the progressive group. Future research may explore the differential responses of men and women to PA interventions and explore the underlying motivational factors influencing engagement, thus enabling the development of tailored interventions that optimize effectiveness for each gender. Notwithstanding these deviations, measures were implemented to guarantee methodological rigor, such as a meticulous random sampling procedure during the recruitment phase, transparent documentation of the transfers, supplementary analysis to assess their potential impacts, and inclusion of gender as a controlled variable in the statistical analysis. Despite significant efforts to reduce the negative effects of this deviation, it is advisable for readers to consider these complexities while interpreting the study’s results.

In a similar vein, if individual differences such as participants’ motivational level have the potential to influence the results of PA interventions, it is important to consider individual and contextual differences as moderators in the relationship between PA protocols and mental health outcomes. These individual and contextual differences may include personality traits [67], self-esteem [68], resilience [69], and group dynamics [70], all of which have been shown to significantly impact mental health indicators. Future research may explore these individual differences as moderators or examine the effects of PA interventions on mental health across various individual differences.

Furthermore, potential biases may arise throughout the recruitment process. For example, selection bias may occur if certain groups of individuals are more likely to engage in a study than others [71]. Individuals with a specific interest in mental health or PA may be more inclined to volunteer, leading to a sample that is not representative. In addition, recruitment bias can occur when some recruitment strategies attract specific demographic groups more than others [72]. For example, web-based advertisements might predominantly reach individuals who frequently visit university-related websites, while distributing flyers at community centers may be more accessible to participants who visit such places more often.

In addition, the inability to monitor participant adherence to the assigned PA protocols represents a potential limitation. Although participants were required to capture and submit their PA records generated by the app, the lack of real-time tracking made it challenging to assess the extent to which participants complied with the prescribed PA protocols. Thus, future studies should implement robust adherence tracking mechanisms to provide a more comprehensive understanding of participant engagement and its relationship to intervention effectiveness.

Finally, this study primarily focused on assessing the PA intervention outcomes in a healthy population. Consequently, the protocols and dosages used in this study may not be directly generalizable to populations with preexisting mental health concerns or diagnoses. Individuals with mental health conditions may have unique needs and considerations when engaging in PA interventions, including tailored exercise prescriptions, supervision, and support mechanisms. Therefore, caution should be exercised when extrapolating the findings of this study to clinical populations, and future research should aim to investigate the efficacy of PA interventions specifically tailored to individuals with mental health concerns.

### Practical Implications

This study underscores the significance of mobile-based PA in generating positive mental health outcomes in adults. Consequently, it is crucial to actively promote and facilitate the adoption of such programs. This can be achieved through diverse approaches, including awareness campaigns, educational materials, and partnerships with fitness professionals or organizations. By emphasizing the convenience and accessibility of mobile-based programs, individuals can be motivated to incorporate PA routines into their daily lives using smartphones or tablets at their preferred times and locations [73].

In order to encourage mobile-based progressive PA programs, governments must engage in efforts such as providing financing or subsidies for the creation and promotion of mobile PA apps. Engaging in collaboration with app developers, fitness experts, and mental health physicians is vital to guarantee the caliber and efficacy of these programs. Furthermore, governments have the capability to incorporate mobile PA programs into public health campaigns, emphasizing their positive impact on mental health and overall well-being.

Finally, fitness facilities might assume a crucial function by collaborating with mobile app developers or developing their own fitness applications. These centers can grant members access to these apps as a component of their membership packages or provide specific programs that integrate in-person instruction with mobile-based PA. Conducting workshops or events to enlighten persons about the advantages of mobile-based progressive PA and enhance their efficacy can also be advantageous.

### Conclusions

This study aimed to fill the existing gap in knowledge regarding the efficacy and application of progressive and fixed PA programs in mobile-based environments, with the ultimate purpose of maximizing mental health advantages. This study produced noteworthy results that provided insights into the influence of this intervention on mental health outcomes. The progressive and fixed PA groups showed a remarkable reduction in depression and perceived stress levels compared with the control group. In addition, only the fixed group showed significant decreases in GAD-7 scores.

These findings offer valuable insights into the potential advantages of engaging in mobile-based PA in enhancing the mental health outcomes of adults. In addition, this study highlights the effectiveness of progressive PA in reducing depressive symptoms and perceived stress, even in mobile-based settings, indicating a specific type of program that yields positive outcomes. Consequently, further development and implementation of innovative strategies that optimize the convenience and effectiveness of mobile-based interventions are warranted to support individuals in achieving better mental health.

## Appendix

Multimedia Appendix 1 CONSORT-eHEALTH checklist (V 1.6.1).

## Abbreviations

BEPSI-K: Brief Encounter Psychosocial Instrument-Korean version
CONSORT: Consolidated Standards of Reporting Trials
GAD-7: Generalized Anxiety Disorder-7
PA: physical activity
PHQ-9: Patient Health Questionnaire-9
RMANOVA: repeated measures ANOVA
